# Leucine-Rich, Potent Anti-Bacterial Protein against *Vibrio cholerae, Staphylococcus aureus* from *Solanum trilobatum* Leaves

**DOI:** 10.3390/molecules27041167

**Published:** 2022-02-09

**Authors:** Manohar Radhakrishnan, Malathy Palayam, Ammar B. Altemimi, Lakshminarayanan Karthik, Gunasekaran Krishnasamy, Francesco Cacciola, Lakshmanan Govindan

**Affiliations:** 1 Center of Advanced Study in Crystallography & Biophysics, University of Madras, Chennai 600025, India; bioinfomalathi@gmail.com (M.P.); lnbioinfokarthik@gmail.com (L.K.); gunaunom@gmail.com (G.K.); 2 Department of Chemistry and Chemical Biology, Indiana University Purdue University, Indianapolis, IN 46202, USA; 3 Department of Plant Biology, University of California, Davis, CA 95616, USA; 4 Department of Food Science, College of Agriculture, University of Basrah, Basrah 61004, Iraq; ammar.ramddan@uobasrah.edu.iq; 5 Department of Biomedical, Dental, Morphological and Functional Imaging Sciences, University of Messina, 98125 Messina, Italy; 6 Sri Lakshmi Narayana Institute of Medical Sciences, Puducherry Affiliated to Bharath Institute of Higher Education and Research, Chennai 605502, India; lakshmanang261988@gmail.com

**Keywords:** *Staphylococcus aureus*, *Vibrio cholerae*, antimicrobial function, *Solanum trilobatum*

## Abstract

A 24 kDa leucine-rich protein from ion exchange fractions of *Solanum trilobatum*, which has anti-bacterial activity against both the Gram-negative *Vibrio cholerae* and Gram-positive *Staphylococcus aureus* bacteria has been purified. In this study, mass spectrometry analysis identified the leucine richness and found a luminal binding protein (LBP). Circular dichroism suggests that the protein was predominantly composed of α- helical contents of its secondary structure. Scanning electron microscopy visualized the characteristics and morphological and structural changes in LBP-treated bacterium. Further in vitro studies confirmed that mannose-, trehalose- and raffinose-treated LBP completely inhibited the hemagglutination ability towards rat red blood cells. Altogether, these studies suggest that LBP could bind to sugar moieties which are abundantly distributed on bacterial surface which are essential for maintaining the structural integrity of bacteria. Considering that *Solanum triolbatum* is a well-known medicinal and edible plant, in order to shed light on its ancient usage in this work, an efficient anti-microbial protein was isolated, characterized and its in vitro functional study against human pathogenic bacteria was evaluated.

## 1. Introduction

Thanks to their constant evolution, many pathogenic bacteria have developed resistance against conventional antibiotics, which has led to a search for alternative therapeutics. In recent times, the use of antimicrobial proteins or peptides has emerged as one of the most reliable options for circumventing the effect of antibiotic-resistant pathogens. In nature, these antimicrobial proteins are present in most species such as insects, plants and mammals as a part of their defense mechanism. They possess diversified functions that range from having actual antimicrobial properties to immunomodulatory effects [1]. Reports have suggested that antimicrobial proteins inactivate prokaryotic cells by targeting essential physiological processes occurring at any of the plasma membrane, extracellular, or intracellular sites [2].

Plants themselves adopt different types of defense mechanisms to protect against microbes, which includes the production of antimicrobial proteins, secondary metabolites, lytic enzymes and membrane-interacting proteins possessing cell wall reinforcement [3]. In the plant defense mechanism, it has been well-documented that leaves have evolved with anti-microbial properties, where, incidentally, leaves from the plants representing the *Solanum* family are widely reported [4,5]. Generally, antimicrobial proteins from plants are low-molecular-weight proteins. Some of them play a role in the immune systems of animals and plants to limit pathogen infection and their growth [6]. Antibacterial proteins from various plants were purified and characterized where most of them exhibited activity towards plant pathogens. The antibacterial proteins have the ability interact with the cell surface of bacteria through specific receptors and collapse the cell membrane [7]. If these proteins are positively charged, they are attracted electrostatically to the negatively charged molecules such as anionic phospholipid, lipopolysaccharide and teichoic acid which are located asymmetrically to the membrane architecture. Consequently, their binding ability to the membrane can activate several pathways that cause cell death. Some antibacterial proteins are pore formers on the membrane and hence work as effectors of innate immunity with special structural features. Moreover, the defensive role is played by different class of proteins by employing different modes of mechanism. Some examples are chitinases, 1,3-glucanases, defensins, thionins, 2S albumins, ribosome-inactivating proteins, lectins and protease inhibitors [8].

*Solanum trilobatum* is a well-known medicinal herb across the Asian continent. Recently, considering its medicinal value, it has gained a lot of popularity and is widely used to treat colds, fevers and throat and lung infections [9]. Other preliminary studies evidenced bioactive compounds were from the leaves’ various extracts with a wide range of pharmacological values. To shed light on its ancient usage, an antimicrobial protein was identified and isolated. *Solanum trilobatum* is used for various ailments in different parts of Asia [10,11] and its active principle sobatum was reported to possess anti-tumor [12,13] anti-inflammatory activities [9]. The tannin of *Solanum trilobatum* leaves are reported to have antibacterial activity [14]. The aqueous extract, methanol extract of the leaves and stem were also reported to have antimicrobial activity. The leaf extract of *Solanum trilobatum* was reported with antioxidant potency [15], oviposition deterrent and repellent activities against the mosquito *Anopheles stephensi* [16]. The presence of resistance gene analogs (RGAS) also been reported in this underexploited plant species.

*Staphylococcus aureus* is a Gram-positive pathogenic organism, and it causes harmful infections on the skin, nasal area and urinary bladder. This organism poses additional complication by its endogenous infection capability to humans and animals. They have also been found to be capable of deactivating immune components of the host and can compete and overcome other common organisms [17]. *Vibrio cholerae* is a Gram-negative human and animal pathogen, which is responsible for small intestine infections that include damage of epithelial cells resulting in diarrhea. From the first infection, in a few hours it can trigger severe *cholerae* leading to dehydration, hypotension and can finally lead to death [18]. Owing to the use of this plant towards solving respiratory problems, indigestion and in search of new anti-bacterial proteins, an attempt has been made to analyze proteins from the aqueous extract of *Solanum trilobatum* leaves. The potent antimicrobial protein identified from *Solanum trilobatum* has been purified and characterized. Furthermore, the possible mechanism of this defensive protein against *Staphylococcus aureus* and *Vibrio cholerae* is described with experimental evidence.

## 2. Results and Discussion

Most of the currently available plant-derived antimicrobial proteins are active against various plant pathogens and against various insects [4]. However, due to lack of purity, none of them were taken for downstream characterization against human pathogens [5]. So, there is an urge to identify new antibacterial principles against such human pathogens, as drug resistance is a major challenging issue faced in the field of medical treatment and diagnosis. To fulfill our needs, we began by testing active principles from *Solanum trilobatum*, a well-known medicinal plant across the Asian continent.

### 2.1. Ammonium Sulfate Fractions of Solanum trilobatum Leaves Are Rich in Antibacterial Action

**Figure 1:**
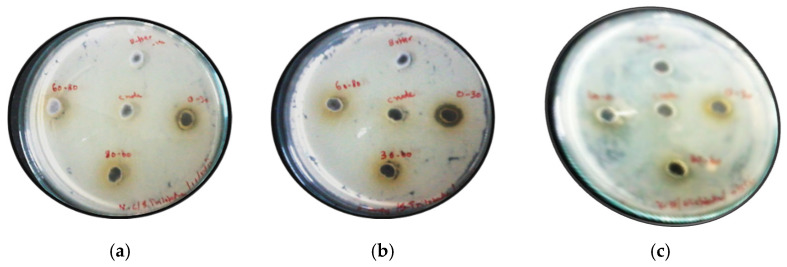
Antibacterial activity of fractionated *Solanum trilobatum* proteins against *Vibrio cholerae*, *Staphylococcus aureus* and *Eshersia coli*, respectively. (**a**–**c**) Centre well—Extracted aqueous crude, Right well—0–30% fraction, Left well—60–80% fraction, Top well—Extraction Buffer (control), Bottom well—30–60% fraction.

The aqueous extract of *Solanum trilobatum* leaves was collected in 20 mM Tris, 150 mM NaCl pH (6.4) buffer, proteins were sorted according to various ammonium sulfate fractions (0–30, 30–60 and 60–80%). The collected protein fractions were dialyzed against 20 mM Tris,150 mM Nacl pH (6.4). The antibacterial activity of each collected fractions, along with a crude extract, were analyzed against both Gram-positive and Gram-negative pathogenic bacteria which includes *Staphylococcus aureus*, *Vibrio cholerae* and *Eschersia coli* using the agar well diffusion method. Interestingly, out of the three collected fractions, the fraction which was salted out at 0–30% saturation of ammonium sulfate showed potent antibacterial activity against *S. aureus* and *V. cholerae* but had no activity against *E. coli* tested bacteria (Figure 1a–c)**.** However, the crude extract collected from the leaves of *Solanum trilobatum* showed zero activity against all the tested bacteria due to some unknown reason. We assumed that the crude extract might be less stable than the active fractions or could express proteolytic enzymes which can degrade the active principle (Figure S1).

### 2.2. Ion Exchange Chromatography of Solanum trilobatum Active Fraction

**Figure 2:**
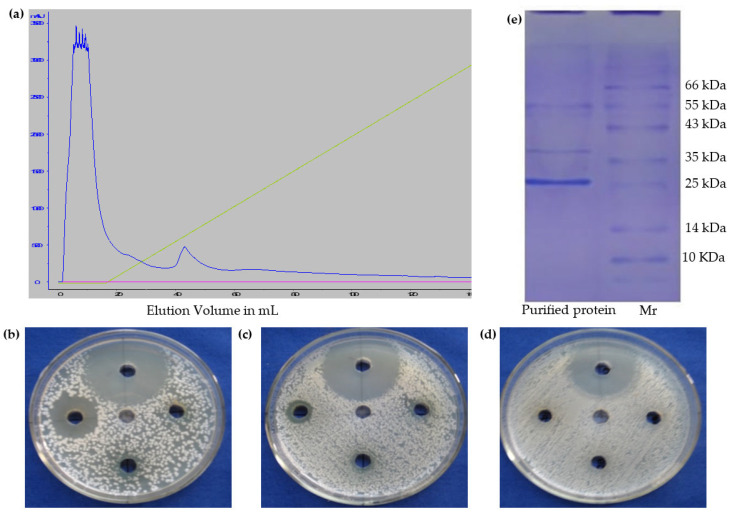
(**a**) Purification of corresponding (0–30%) active antibacterial fraction by ion exchange chromatography (linear gradient from c88150 mM to 1 M NaCl). (**b**–**d**) Antibacterial activity of bound fraction vs. *Vibrio cholerae*, *Staphylococcus aureus* and *Eshersia coli*, respectively. Top well—100 µg of Ampicilin (Control), Centre well—100 µg of unbound fraction, Right well—25 µg of bound fraction, Bottom well—50 µg of bound fraction, Left well—100 µg of bound fraction. (**e**) Purified *Solanum trilobatum* antibacterial protein (left lane) on 15% Poly acrylamide gel electrophoresis with standard marker (right lane).

To purify the active principle, 0–30% fraction was subjected to ion-exchange chromatography using DEAE-Sepharose column by liner gradient elution mode. The purification yielded two major peaks which were an unbound peak collected at 10th and the bound elutions at min 42 (active principle) of the run (Figure 2a). Both the bound and unbound fractions were tested for their anti-bacterial activity against *S. aureus*, *V. cholerae* and *E. coli* along with ampicillin (positive control); the bound fraction showed potent antibacterial activity at all the tested concentrations against *S. aureus* and *V. cholerae* bacteria (Figure 2b,c) but had zero activity against *E. coli*. To determine the minimum inhibitory concentration, the prepared 1 mg/mL of purified protein by broadford method were serially diluted up to 10 µg by microdilution. Both the bacteria were treated with the diluted protein with ampicillin as control, MIC were determined based on the turbidity that observed at 460 nm were measured by Epoch plate reader. Where at 10, 20 µg of protein concentration the turbidity could be observed, but at 30 µg it could not be observed due to antibacterial action on both the bacteria. So, MIC of the protein is found to be 30 µg/mL towards both Gram-positive and Gram-negative bacteria (Table 1).

### 2.3. The Antibacterial Protein Isolated from the Leaves of Solanum trilobatum Is a Leucine-Rich Luminal Binding Protein (LBP)

For confirming the purity of ion exchange fraction, the sample was analyzed on 15% SDS-PAGE [19], which confirmed that the fraction corresponds to a protein with a molecular weight of approximately 24 kDa in size. (Figure 2e). For further identification, the desired band was excised from the SDS gel and was subjected to in-gel trypsin digestion, followed by MALDI-TOF-MS/MS analysis. The identified peptides were analyzed using MASCOT algorithm across available plat data base in Swissport. Interestingly, the protein was highly rich in amino acid leucine (Table 2), and the MASCOT search revealed that eight identified peptides were matched to the sequence of the luminal binding protein of *Nicotiana tabucum* 8 (Supplementary Figure S2). Interestingly, the analogue of LBP, otherwise known as the endoplasmic reticulum (ER)-resident luminal binding protein (LBP), is well known for various stress-associated responses in plants. It has been shown to confer drought tolerance when constitutively overexpressed in *Nicotiana* *tabacum* [20]. Even in animal systems, LBP has been proved as a multifunctional protein with numerous functions [21]. LBP assists in protein folding and involves in the ER quality regulatory mechanism which recognizes misfolded proteins and results in selective degradation of such unfolded proteins [21,22]. It was also reported that overexpression of LBP in mammalian cultured cells [23] and tobacco protoplasts increases tolerance to ER stress [24]. LBP regulates immunity mediated by Xa3/Xa26 and Xa 21 against bacterial pathogens *Xanthomonas oryzae* and fungal pathogen *Magnaporthe oryzae* [25]. Although the role of LBP is well characterized in plants and animals, in the present study, for the first time, we reported its role as an antibacterial agent against human pathogens.

### 2.4. LBP Is Majorly Composed of α Helix

**Figure 3:**
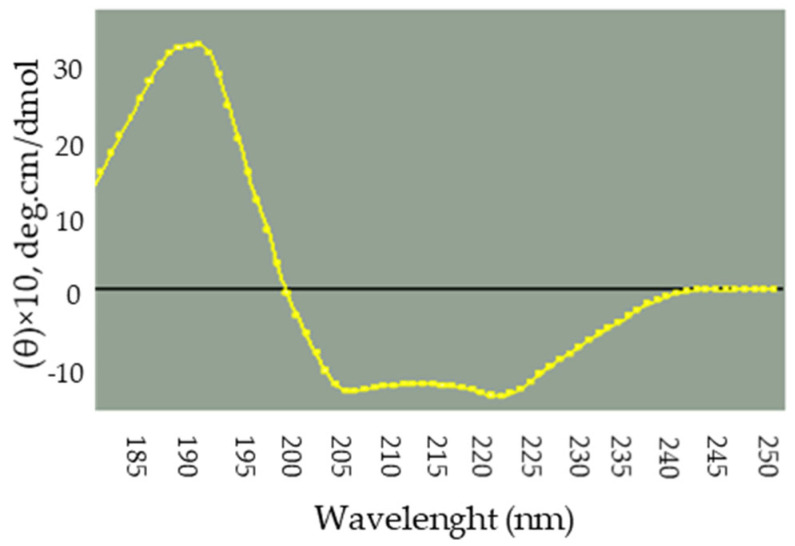
Secondary structure content of LBP, indicating the richness of α-helical content.

Majority of the plant derived antibacterial proteins are rich in helical conformation [26,27,28,29]. In order to gain more knowledge about the structural aspect of *Solanum trilobatum* LBP, we performed a Far UV CD spectrum of using purified LBP at 25 °C and analyzed a wavelength ranging from 190 to 240 nm. The spectrum clearly suggested the existence of positive peak (192 nm) and a negative peak (222 nm), respectively. This clearly evidenced that LBP is rich in characteristic α helix. This was cross-verified by K2D2 [30]. The analysis displayed that LBP is composed of 70% of alpha helices and remaining part composed of beta strands and coils (Figure 3). We assumed the higher helicity could be a possible reason which resulted in forming pores across bacterial membrane.

### 2.5. Molecular Modelling of LBP

**Figure 4:**
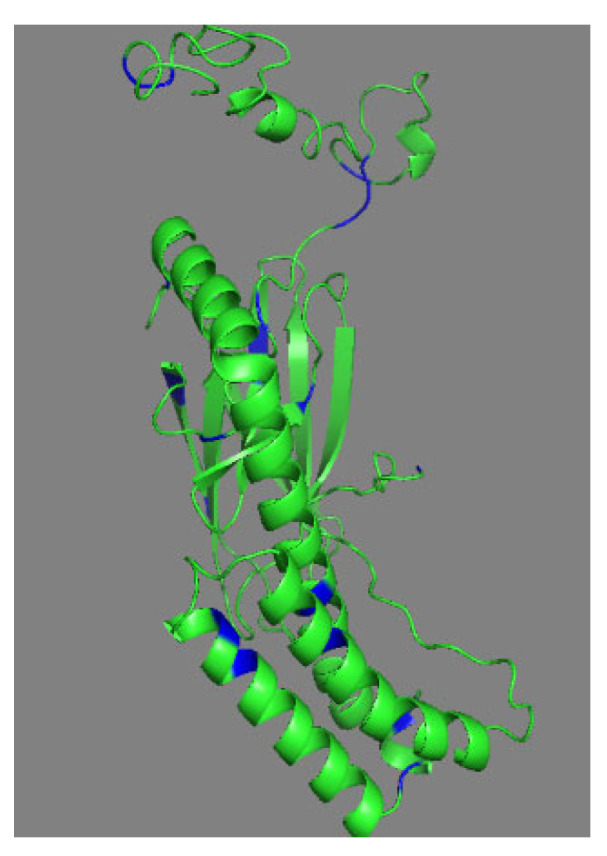
Swiss model generated LBP model based on template (PDB: 7KZI) with 50% of sequence Identity.

Attempts were made to deduce the 3-D structure of the antimicrobial protein (LBP) using homology modeling. The amino acid sequence that was obtained from the Mass Spec result was submitted in Swiss Model and I-TASSER servers using PDB id: 7KZI was used as model template. Where the obtained model composed of majorly composed of helices correlates (Figure 4) and leucine residues were highlighted in blue color.

### 2.6. LBP Treatment Imparts Characteristic Morphological Changes in V. cholerae and S. aureus

**Figure 5:**
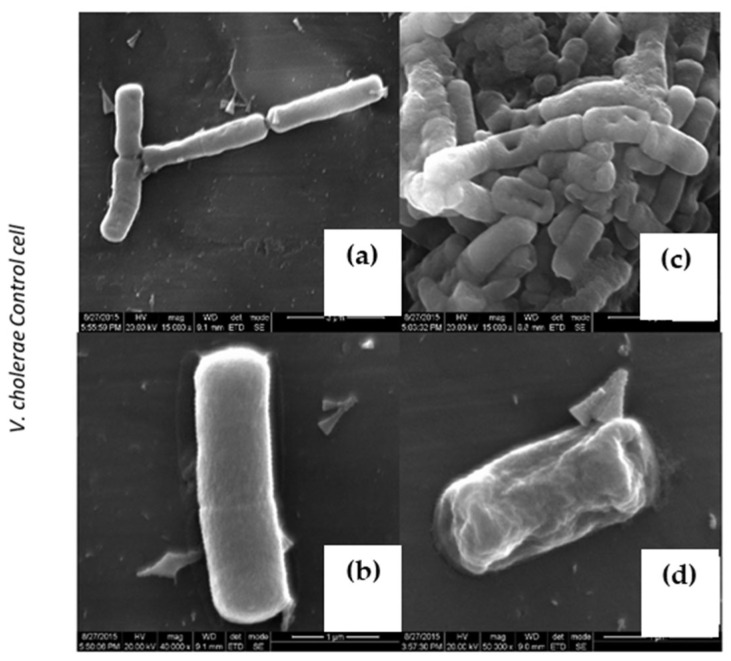
Characteristic morphological changes in *V. cholerae*. (**a**,**b**) Untreated cells. (**c**) Treated with LBP at Sub MIC (30 μg/mL), (**d**) Treated with at Sup MIC (60 μg/mL).

**Figure 6:**
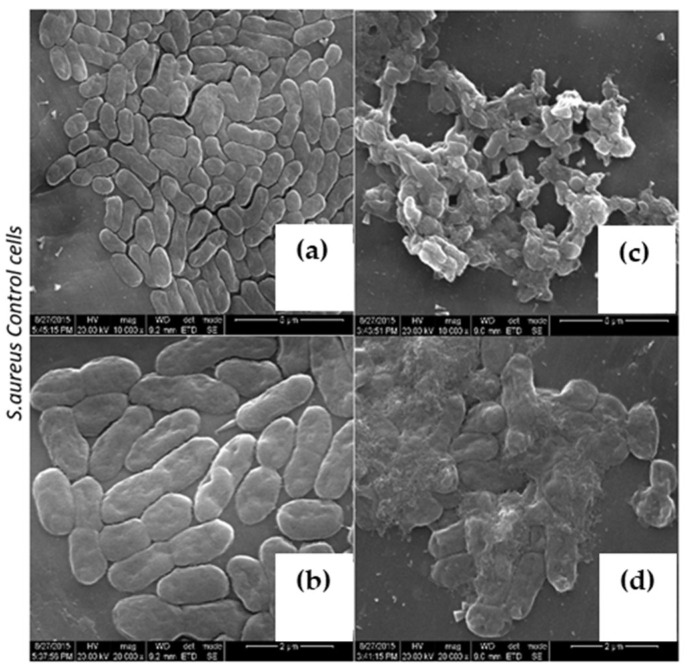
Characteristic morphological changes on *S. aureus*. (**a**,**b**) Untreated cells. (**c**) Treated with LBP at Sub MIC (30 μg/mL) (**d**) Treated with LBP at Sup MIC (60 μg/mL).

**Figure 7:**
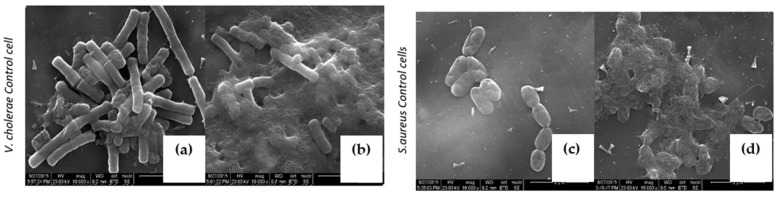
Upon higher level treatment of LBP against *V. cholerae* and *S. aureus*. (**a**,**c**) Untreated *V. cholerae* and *S. aureus* cells. (**b**,**d**) 100 μg/mL treatment of LBP on *V. cholerae* and *S. aureus* respectively.

To explore the mode of action on various pathogens, *V. cholera* and *S. aureus* cultures were treated with 30 μg/mL concentrations and above MIC of purified LBP and were subjected to a scanning electron microscopic analysis (SEM). Interestingly, LBP-treated bacterium exhibits characteristic morphological changes. Characteristic thinning of bacilli were evident on both *V. cholera* and *S. aureus* upon LBP treatment (Figure 5d and Figure 6c,d), and more importantly bacterium was aggregated upon LBP treatment, which suggests that LBP might potentially act on the bacterial membrane or cell wall. At the same time, we also observed distinguished pore formation on the bacterial membrane upon LBP treatment at sub MIC (Figure 5c) such as protein extracted from mucus of fish [31], toxin α-hemolysin [32] and which is characteristic of various available beta lactam classes of antibiotics [33]. These unique features play a major role in industrial biotechnology antibiotic production. At a higher concentration treatment of LBP, both cell membranes were disintegrated and rupture was witnessed (Figure 7c,d).

### 2.7. LBP Had Agglutination on Rat RBCs

**Figure 8:**
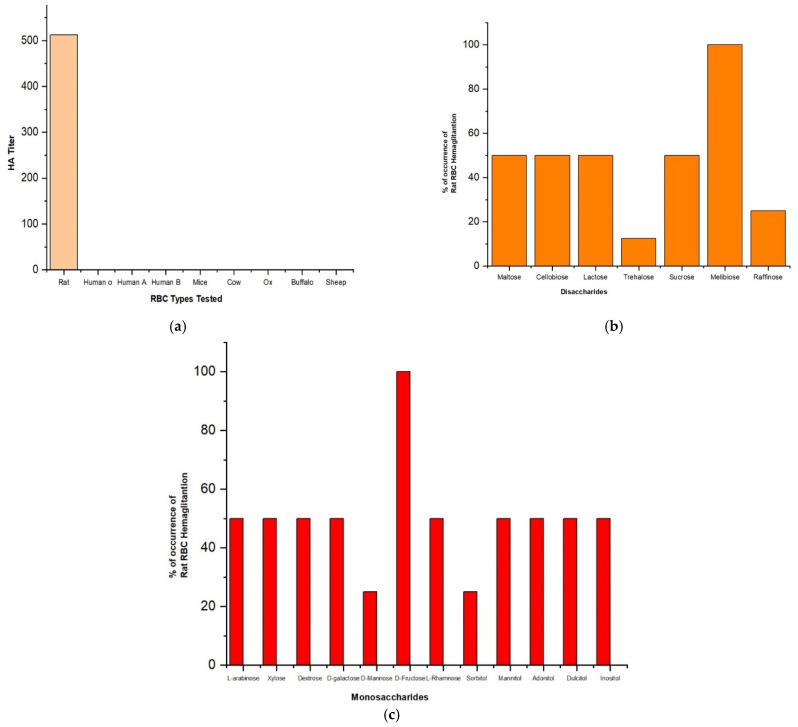
(**a**) Hemagglutination (HA) activity of LBP against various mammalian RBC types. (**b**,**c**) Carbohydrate inhibition assay on Rat-RBC hemagglutination activity of LBP.

To understand the self-lysing activity of LBP on human RBS, we performed a hemagglutination assay using RBCs isolated from different mammals: rodents. Interestingly, LBP expressed high hemolysis activity in the tested Rat RBCs but had zero activity across all the other mammalian activity (Figure 8a); this justifies the edibleness for higher mammalians and many useful properties of the raw leaves of *Solanum trilobatum*, as it does not interact with the human blood cells and other higher mammalians such as cow, ox, buffalo, and sheep RBSs.

### 2.8. LBP Binds to Sugars Such as Trehalose, Mannose, Raffinose and Sorbitol

Since distinct morphological changes were evident on bacterial surface up on LBP treatment, as bacterial membrane is rich is various kinds of sugars [34], we assumed that LBP could bind to various sugar moieties on bacterial membranes in order rupture its morphology. Towards this, we incubated purified LBP with various mono-, di- and polysaccharides and its hemagglutination ability on rat RBSs was confirmed using HA titer assay. We observed reduced rat RBCs hemagglutination upon incubation with trehalose, mannose, sorbitol and raffinose (Figure 8b,c). This result suggests that LBP could interact with these sugars which are abundantly distributed on bacterial membrane to perform its antibacterial activity [35].

## 3. Materials and Methods

### 3.1. Bacterial Cultures and Reagents Used in Present Study

*Vibrio cholerae* (3906)*, Staphylococcus aureus* (96) and *Escherichia coli* (1687) were purchased from MTCC Culture Bank (Institute of Microbial Technology, Shanti Path, Chandigarh, India). Diethoxy aminoethyl Sepharose-5 mL Column was purchased from GE Life Sciences (Bangalore, India), whereas all other chemicals which are mentioned in the present study were purchased from MilliporeSigma (Bedford, MA, USA).

### 3.2. Extraction of Principle Isolate from Solanum trilobatum

The leaves of *Solanum trilobatum* were separated from the thorn, washed, and blended in a mortar and pestle in 20 mM Tris, 150 mM NaCl pH (6.4) buffer. The crude extract was filtered and centrifuged at 12,000 rpm for 30 min at 4 °C. Finally, the clear supernatant was retained. To perform, ammonium sulphate fractionation, initially a 0–30% relative saturation was attained [36]. The precipitate formed after standing for 1 h at 4 °C was collected by centrifugation at 12,000 rpm for 30 min in 4 °C and the pellet of each gram was dissolved with the 3 mL of the 20 mM Tris, 150 mM Nacl pH (6.4) buffer. Similarly, 30–60 and 60–80% precipitations were performed accordingly. All the three fractions were dialyzed using 10 kDa cut off dialysis membrane against 20 mM Tris, 150 mM NaCl pH (6.4) buffer at 4 °C over night and further for 4 h with freshly prepared buffer.

### 3.3. Antibacterial Assay by Well Diffusion Method

The individual colonies of the *Staphylococcus aureus, Vibrio cholerae* and *Escherichia coli* were picked from the prepared glycerol stock, and they were allowed to grow in 3 mL of autoclaved LB broth at 37 °C overnight. Luria-Bertani agar medium was prepared and poured in to the sterile 90 mm-diameter petri plates. The agar medium was allowed to solidify at ambient temperature. The bacteria cultures were spread on surface of the petri plates using sterile swab. Wells were made using a sterilized punch rod and the samples which were to be analyzed were added into the corresponding wells using a sterile micropipette. The inoculated plates were initially kept undisturbed for 15 min in the laminar flow at room temperature, and then they were shifted, incubated at 37 °C for 24 h and the plates were examined for antibacterial activity [37].

### 3.4. Purification of Antibacterial Protein by Ion-Exchange Chromatography

To purify the antibacterial protein, the active fraction (0–30%) was subjected to ion exchange chromatography. The active fraction was loaded on to 5 mL of weak anionic prepacked DEAE (Hi-Trap—5 mL) column. The column was pre-equilibrated with 20 mM Tris, 150 mM NaCl pH (6.4) buffer. The unbound proteins were collected by wash buffer (20 mM Tris, 150 mM NaCl pH (6.4)) about 4 column volume (CV), and the bound proteins were eluted about 25 CV by linear gradient of 1 M NaCl with a flow rate of 1 mL/min. Both the unbound and bound fractions were subjected to further antibacterial activity [38].

### 3.5. Minimal Inhibitory Concentration (MIC) by Micro Dilution Assay

MICs were determined based on the procedure of National Committee for Clinical Laboratory Standards (NCCLS) and Clinical and Laboratory Standard Institute (CLSI). Luria Bertani Broth (LHB) was used as the main assay medium for the tested organisms *Staphylococcus aureus* and *Vibrio cholerae*. Freshly grown cultures at exponential phase were used as the inoculums. Bacteria were centrifuged at 4000 rpm for 15 min at 4 °C, suspended in LB broth and adjusted to 4 × 10^6^ C.F.U/mL [39]. The active fraction containing pure protein was prepared to 1 mg/mL in 20 mM Tris, 150 mM NaCl pH (6.4) buffer and was used as sock solution. Protein solution at different concentrations ranging from 10 to 100 µg were added to each bacterium well of a 96-well, flat-bottomed plate (Tarson) and made up to 100 µL using 20 mM Tris, 150 mM NaCl pH (6.4) buffer. Plates were incubated at 37 °C for 24 h. The experiment was carried out in triplicate. The MIC was defined as the lowest concentration of the protein that prevented visible turbidity, as measured at 460 nm by using an ELISA microplate reader (mod. Epoch, BioTek Instruments, Vermont, VT, USA). Visible turbidity was determined by the OD of tested samples that were significantly greater than that of the medium, i.e., background [40]. The susceptibility study was compared with 20 mM Tris, 150 mM NaCl pH (6.4) buffer and ampicillin as negative and positive controls respectively.

### 3.6. Mass Spectrometry—Peptide Mass Fingerprinting

The purified 24 kDa protein was excised from Coomassie blue R-250 stained SDS Polyacrylamide gel and digested by trypsin as per previous reports [41]. The trypsinized protein was loaded onto a mass spectrometer (Bruker, MALDI-TOF/TOF). The obtained peptide peaks (Bio Tools version 2.2 software Bruker Daltonics, Avalilable online: https://bruker-daltonics-biotools.software.informer.com/2.2/, accessed on 14 December 2021) and their corresponding masses were analyzed by the MASCOT engine search tool. All were compared to the NCBI database (Matrix Science Inc., Boston, MA, USA) [42]. The results were statistically analyzed by one-way analysis of variance (ANOVA) using the software SPSS 13 (SPSS Inc., Chicago, IL, USA), validated and possible match peptide fragment sequences were taken.

### 3.7. Circular Dichroism Measurements to Analyze the Secondary Structural Content of STAP

The CD measurements of the purified protein were carried out using Jasco J 815 polarimeter. The instrument was calibrated with a standard solution of (+)-10-camphorsulfonic acid. The measurements were recorded with 0.1 mg/mL of purified antibacterial protein in wavelength range of 190–240 nm at 25 °C at a resolution of 1 nm using Quartz cuvettes of 0.1 cm path length (Hellma, New York, NY, USA). Data were collected as triplets and the average spectrum has been taken for processing after baseline correction with the buffer spectrum. K2D2 software (Estimation of protein secondary structure from CD spectra. Avalilable online: http://cbdm-01.zdv.uni-mainz.de/~andrade/k2d2/, accessed on 18 December 2021) was used for data analysis. Mean Residue Ellipticity was calculated and utilized for secondary structure determination [43].

### 3.8. Homology Modelling of S

Sequence analysis performed using pBLAST for the identified eight peptide fragments of the purified protein. The found fragments covered 33% of sequence of *Nicotiana tabcum*. The obtained sequence shares 50% of structural similarity with PDB id:7KZI. A homology model was constructed with Swiss Modeler (http://swissmodel.expasy.org/, accessed on 27 November 2021), I TASSER (https://zhanggroup.org/I-TASSER, accessed on 27 November 2021) [44,45], (PDB id:7KZI) as a template. The final homology model was visualized on Pymol viewer (PyMOL Molecular Graphics System, Version 2.0 Schrödinger, LLC, New York, NY, USA) [46]. The quality of the model was estimated based on C score—1.01 (confidant of score in I-TASSER). 

### 3.9. Scanning Electron Microscope (SEM) Studies on STAP

Five milliliters of optimally grown *Staphylococcus aureus* and *Vibrio cholerae* were treated with purified proteins of respective MIC concentrations and were incubated at 37 °C for 24 h in an orbital shaker at 120 rpm. The pellets which were obtained after centrifugation at 4000 rpm for 20 min were washed with 1.0 mL of phosphate buffer saline (PBS, pH 7.2). The pellets were fixed with glutaraldehyde (2.8%) and incubated for 2 h at room temperature. After removal of unreacted glutaraldehyde by washing and centrifugation, once again the pellet was incubated for 15 min at room temperature in PBS. The postfixed specimens were dehydrated with 30% ethanol [47]. The cells were allowed to dry before sputter coating for SEM analysis (HITACHI H-7650, Tokyo, Japan). Ultra-structural changes induced by the antibacterial protein on bacterial cell wall were recorded.

### 3.10. Hemagglutination Assay

Human blood samples (A, B and O blood groups) were obtained from a blood bank, Stanley Medical College, Chennai, India. Sheep, goat, ox, cow and buffalo blood samples were collected from the Corporation Slaughterhouse, Perambur, Chennai. Rabbit, rat and mouse blood samples were received as a gift from control animals maintained in our laboratory by venous or cardiac puncture. The hen blood cells were collected from a chicken shop, Kotturpuram, Chennai. All blood samples were collected in Alsever’s solution, stored at 10 °C and used within 5 days [48]. The collected blood samples were washed thrice with 0.9% physiological saline and once with TBS by centrifugation for 10 min at 8000 rpm. Unless otherwise specified, the washed erythrocyte pellets were finally resuspended in TBS to make up to 1.5% (*v/v*) erythrocytes suspension.

#### Alsever’s Solution

Alsever’s solution was prepared according to the reported method [49]. Briefly, 10.25 g dextrose, 4 g tri-sodium citrate, 0.28 g citric acid and 2.10 g of sodium chloride were dissolved in 500 mL of double distilled water. The solution was autoclaved, cooled to room temperature and then 50 mg of streptomycin was dissolved in this solution and stored at 10 °C until use.

The hemagglutination (HA) assays which were routinely performed in V-bottom microtiter plates (Tarsons) by serial dilution of a 25 µL sample with an equal volume of appropriate buffer (TBS). Under certain experimental conditions, TBS without any divalent cation was used for HA. After dilution, 25 µL of RBC suspension (1.5% *v/v*) was added to each well and incubated for 45 min at 25 °C. The hemagglutination titer was recorded as reciprocal of the highest dilution of the sample causing complete agglutination of RBCs [50]. In controls, buffer was substituted for sample. Each experiment was performed in duplicate three to five times using samples from different preparations, and the agglutinating activity of the test sample was analyzed based on the median agglutination titer values obtained for each RBC type.

### 3.11. Hemagglutination-Inhibition Assays

To characterize hemagglutination activity of the antibacterial protein, inhibition assays were carried out to find out the role of carbohydrates. Carbohydrates (stock solutions at 200 mM) were prepared in Tris Buffer Saline (TBS) (50 mM Tris pH 7.4, 115 mM NaCl, 305 mOsm) and tested for their ability to inhibit the HA activity in extract against the RBC types. The pH of these test solutions were adjusted to 7.4 using 1 M NaOH.

#### Carbohydrates Binding Specificity of LBP

The role of sugars in hemagglutination and their specificity towards Rat RBC were estimated using various carbohydrates. The purified protein was first diluted with TBS pH (7.4) buffer to provide a hemagglutination titer of 4 against Rat RBC. The diluted protein (25 µL) was added to each well of the microtiter plate, then 25 µL of each test carbohydrate solution was added to the first well and serially diluted up to 7 wells and the least well was kept as the control in microtiter plates and incubated for 1 h at 25 °C. After incubation, 25 of 1.5% RBC suspension was added to each well, incubated for 45 min at 25 °C and occurrence of hemagglutination in each well was carefully observed. The minimal concentration of the test substance that completely inhibited the hemagglutinating activity was recorded [51].

## 4. Conclusions

This work reports the functional aspects of an antibacterial protein from the leaves of *Solanum trilobatum*, a plant which is commonly used to recover from respiratory, intestinal and throat infections, where respiratory and intestinal tract infections were mainly reasonable of *S. aureus*. To characterize further morphological changes on the shape and surface of bacterial cells, the purified-protein-treated and -untreated cells were analyzed through scanning electron microscope. Both Gram-positive and -negative microbes underwent significant rupturing of their cell walls, thereby suffering from cell content leakage. That said, although the protein possesses agglutination activity on both bacteria, pore formation causes quick damage to *Vibrio cholerae*, which has been witnessed. In short, total lysis of the cell membrane was witnessed against both Gram-negative (*V. cholerae*) and -positive (*S. aureus*) bacteria. Major helical content and hemagglutination and its inhibition by trehalose, mannose, sorbitol and raffinose could be pointed out as important properties by which the protein could bind or interact with the cell membrane receptors and cause cell lysis. In addition, the inability of it to agglutinate human blood validates its edibleness. The protein was identified as Leucine-rich luminal binding protein by peptide mass fingerprinting, and we modelled the three-dimensional structure. This is the first report of an antibacterial protein from *Solanum trilobatum* leaves, and its action against *V. cholerae* and *S. aureus* has been reported.

## Figures and Tables

**Table 1:** M.I.C determination. The reading at 460 nm was taken in Epoch Elisa Microplate reader. + Turbidity observed, − Turbidity not observed.

Tested Strain	Different Protein Concentrations Tested (1 mg/mL)									
	10 μL	20 μL	30 μL	40 μL	50 μL	60 μL	70 μL	80 μL	90 μL	100 μL
*S. aureus*	+	+	−	−	−	−	−	−	−	−
*V.cholerae*	+	+	−	−	−	−	−	−	−	−
Negative Control	+	+	+	+	+	+	+	+	+	+
Positive Control	−	−	−	−	−	−	−	−	−	−

**Table 2:** Obtained peptide fragments of *Solanum trilobatum* antibacterial protein indicates the leucine richness.

No. of Fragments	Observed	Mr Expt	Obtained Fragments
2–10	1066.6100	1065.6027	IPKVQQ**LL**K
2–16	1791.9340	1790.9267	IPKVQQ**LL**KDYFDGK
49–71	2384.2130	1790.9267	DI**LLL**DVAP**L**T**L**GIETVGGVMTK
72–83	1365.7900	1364.7827	**L**IPRNTVIPSKK
84–106	2705.4460	2704.4387	SQVFTTYQDQQTTVTIQVFEGER
118–128	1157.7100	1156.7027	FDLTGIAPAPR
177–189	1554.8060	1553.7987	MVKEAEEFAEEDK

## Data Availability

Not applicable.

## Supplementary Materials

The following are available online. Figure S1. Proteolytic activity of solanum trilobatum aqueous extract where Casein was used as substrate by agar welldiffusion method: Top well—*Solanum trilobatum* (Crude extract), Bottom well—Protein Extraction buffer (20 mM Tris Buffer pH (6.4), Left well—Distilled water, Right well—150 mM NaCl. Figure S2. Obtained peptide fragments of *Solanum trilobatum* antibacterial protein from Mass Spectrometer, identified the leucine richness and eight fragments, those fragments were matched with luminol binding protein of *Nicotiana tabacum*.

## Sample Availability

Samples of the compounds are available from the authors.

## References

[1] Choi K.-Y., Chow L.N., Mookherjee N. (2012). Cationic host defence peptides: Multifaceted role in immune modulation and inflammation. J. Innate Immun..

[2] Yount N.Y., Yeaman M.R. (2013). Peptide antimicrobials: Cell wall as a bacterial target. Ann. N. Y. Acad. Sci..

[3] Gonzalez-Lamothe R., Mitchell G., Gattuso M., Diarra M.S., Malouin F., Boouarab K. (2009). Plant antimicrobial agents and their effects on plant and human pathogens. Int. J. Mol. Sci..

[4] Feng J., Yuan F., Gao Y., Liang C., Xu J., Zhang C., He L. (2003). A novel antimicrobial protein isolated from potato (*Solanum tuberosum*) shares homology with an acid phosphatase. Biochem. J..

[5] Chowdhury N., Laskar S., Chandra G. (2008). Mosquito larvicidal and antimicrobial activity of protein of *Solanum villosum* leaves. BMC Complement. Altern. Med..

[6] Campos M.L., de Souza C.M., de Oliveira K.B.S., Dias S.C., Franco O.L. (2018). The role of antimicrobial peptides in plant immunity. J. Exp. Bot..

[7] Pelegrini P.B., Del Sarto R.P., Silva O.N., Franco O.L., Grossi-De-Sa M.F. (2011). Antibacterial peptides from plants: What they are and how they probably work. Biochem. Res. Int..

[8] Manohar R., Kutumbarao N.H.V., Nagampalli R.S.K., Velmurugan D., Gunasekaran K. (2018). Structural insights and binding of a natural ligand, succinic acid with serine and cysteine proteases. Biochem. Biophys. Res. Commun..

[9] Emmanuel S., Ignacimuthu S., Perumalsamy R., Amalraj T. (2006). Antiinflammatory activity of *Solanum trilobatum*. Fitoterapia.

[10] Govindan S., Viswanathan S., Vijayasekaran V., Alagappan R. (1999). A pilot study on the clinical efficacy of *Solanum xanthocarpum* and *Solanum trilobatum* in bronchial asthma. J. Ethnopharmacol..

[11] Balu S., Madhavan S., Babu T. (1996). Effect of foliar fertilisation on growth of *Solanum trilobatum* L.. Anc. Sci. Life..

[12] Mohanan P.V., Devi K.S. (1996). Cytotoxic potential of the preparations from *Solanum trilobatum* and the effect of sobatum on tumour reduction in mice. Cancer Lett..

[13] Shahjahan M., Sabitha K.E., Jainu M., Devi C.S.S. (2004). Effect of *Solanum trilobatum* against carbon tetrachloride induced hepatic damage in albino rats. Indian J. Med. Res..

[14] Doss A. (2009). Preliminary phytochemical screening of some Indian Medicinal Plants. Anc. Sci. Life.

[15] Shahjahan M., Vani G., Shyamaladevi C.S. (2005). Effect of *Solanum trilobatum* on the antioxidant status during diethyl nitrosamine induced and phenobarbital promoted hepatocarcinogenesis in rat. Chem. Biol. Interact..

[16] Rajkumar S., Jebanesan A. (2005). Oviposition deterrent and skin repellent activities of *Solanum trilobatum* leaf extract against the malarial vector *Anopheles stephensi*. J. Insect Sci..

[17] Jenkins A., Diep B.A., May T.T., Vo N.H., Warrener P., Suzich J., Stover C.K., Sellman B.R. (2015). Differential expression and roles of *Staphylococcus aureus* virulence determinants during colonization and disease. mBio.

[18] Faruque S.M., Albert M.J., Mekalanos J.J. (1998). Epidemiology, genetics, and ecology of toxigenic *Vibrio cholerae*. Microbiol. Mol. Biol. Rev..

[19] Laemmli U.K. (1970). Cleavage of structural proteins during the assembly of the head of bacteriophage T4. Nature.

[20] Alvim F.C., Carolino S.M.B., Cascardo J.C.M., Nunes C.C., Martinez C.A., Otoni W.C., Fontes E.P.B. (2001). Enhanced accumulation of BiP in transgenic plants confers tolerance to water stress. Plant Physiol..

[21] Ma Y., Hendershot L.M. (2004). ER chaperone functions during normal and stress conditions. J. Chem. Neuroanat..

[22] Malhotra J.D., Kaufman R.J. (2007). The endoplasmic reticulum and the unfolded protein response. Semin. Cell Dev. Biol..

[23] Morris J.A., Dorner A.J., Edwards C.A., Hendershot L.M., Kaufman R.J. (1997). Immunoglobulin binding protein (BiP) function is required to protect cells from endoplasmic reticulum stress but is not required for the secretion of selective proteins. J. Biol. Chem..

[24] Leborgne-Castel N., Jelitto-Van Sooren E.P., Crofts A.J., Denecke J. (1999). Overexpression of BiP in tobacco alleviates endoplasmic reticulum stress. Plant Cell.

[25] Park C.-J., Song M.-Y., Kim C.-Y., Jeon J.-S., Ronald P.C. (2014). Rice BiP3 regulates immunity mediated by the PRRs XA3 and XA21 but not immunity mediated by the NB-LRR protein, Pi5. Biochem. Biophys. Res. Commun..

[26] Zhang Z., Thomma B.P. (2013). Structure-function aspects of extracellular leucine-rich repeat-containing cell surface receptors in plants. J. Integr. Plant Biol..

[27] Batista A.B., Oliveira J.T.A., Gifoni J.M., Pereira M.L., Almeida M.G.G., Gomes V.M., Da Cunha M., Ribeiro S.F.F., Dias G.B., Beltramini L.M. (2014). New insights into the structure and mode of action of Mo-CBP3, an antifungal chitin-binding protein of Moringa oleifera seeds. PLoS ONE.

[28] Shin S.Y., Kang J.H., Hahm K.S. (1999). Structure-antibacterial, antitumor and hemolytic activity relationships of cecropin A-magainin 2 and cecropin A-melittin hybrid peptides. J. Pept. Res..

[29] Dathe M., Wieprecht T. (1999). Structural features of helical antimicrobial peptides: Their potential to modulate activity on model membranes and biological cells. Biochim. Biophys. Acta.

[30] Perez-Iratxeta C., Andrade-Navarro M.A. (2008). K2D2: Estimation of protein secondary structure from circular dichroism spectra. BMC Struct. Biol..

[31] Ebran N., Julien S., Orange N., Saglio P., Lemaitre C., Molle G. (1999). Pore-forming properties and antibacterial activity of proteins extracted from epidermal mucus of fish. Comp. Biochem. Physiol. Part A Mol. Integr. Physiol..

[32] Panchal R., Smart M.L., Bowsed D.N., Williams D.A., Petrou S. (2002). Pore-forming proteins and their application in biotechnology. Curr. Pharm. Biotechnol..

[33] Page M.I. (1984). The mechanisms of reactions of beta-lactam antibiotics. Acc. Chem. Res..

[34] Caroff M., Karibian D. (2003). Structure of bacterial lipopolysaccharides. Carbohydr. Res..

[35] Zentella R., Mascorro-Gallardo J.O., Van Dijck P., Folch-Mallol J., Bonini B., Van Vaeck C., Gaxiola R., Covarrubias A.A., Nieto-Sotelo J., Thevelein J.M. (1999). A *Selaginella lepidophylla* trehalose-6-phosphate synthase complements growth and stress-tolerance defects in a yeast *tps1* mutant. Plant Physiol..

[36] Wingfield P. (1998). Protein precipitation using ammonium sulfate. Curr. Protoc. Protein Sci..

[37] Allen K.L., Molan P.C., Reid G.M. (2011). A Survey of the Antibacterial Activity of Some New Zealand Honeys. J. Pharm. Pharmacol..

[38] Jungbauer A., Hahn R. (2009). Ion-exchange chromatography. Methods Enzymol..

[39] Fuchs P.C., Barry A.L., Brown S.D. (1998). In vitro antimicrobial activity of MSI-78, a magainin analog. Antimicrob. Agents Chemother..

[40] Andrews J.M. (2001). Determination of minimum inhibitory concentrations. J. Antimicrob. Chemother..

[41] Jensen O.N., Podtelejnikov A., Mann M. (1996). Delayed extraction improves specificity in database searches by matrix-assisted laser desorption/ionization peptide maps. Rapid Commun. Mass Spectrom..

[42] Williams K.R., Stone K.L. (1995). In gel digestion of SDS PAGE-separated proteins: Observations from internal sequencing of 25 proteins. Tech. Protein Chem..

[43] Whitmore L., Wallace B.A. (2008). Protein secondary structure analyses from circular dichroism spectroscopy: Methods and reference databases. Biopolym. Orig. Res. Biomol..

[44] Boeckmann B., Bairoch A., Apweiler R., Blatter M.-C., Estreicher A., Gasteiger E., Martin M.J., Michoud K., O’Donovan C., Phan I. (2003). The SWISS-PROT protein knowledgebase and its supplement TrEMBL in 2003. Nucleic Acids Res..

[45] Yang J., Yan R., Roy A., Xu D., Poisson J., Zhang Y. (2015). The I-TASSER Suite: Protein structure and function prediction. Nat. Methods.

[46] De Lano W.L. (2002). The PyMOL Molecular Graphics System.

[47] Hartmann M., Berditsch M., Hawecker J., Ardakani M.F., Gerthsen D., Ulrich A.S. (2010). Damage of the bacterial cell envelope by antimicrobial peptides gramicidin S and PGLa as revealed by transmission and scanning electron microscopy. Antimicrob. Agents Chemother..

[48] Johnson H.M., Brenner K., Angelotti R., Hall H.E. (1966). Serological studies of types A, B, and E botulinal toxins by passive hemagglutination and bentonite flocculation. J. Bacteriol..

[49] Bukantz S.C., Rein C.R., Kent J.F. (1946). Studies in complement fixation; preservation of sheep’s blood in citrate dextrose mixtures (modified Alsever’s solution) for use in the complement fixation reaction. J. Lab. Clin. Med..

[50] Garvey J., Cremer N., Sussdorf D. (1979). Methods in Immunology.

[51] Johnson H.M., Brenner K., Hall H.E. (1966). The use of a wate-soluble carbodiimide as a coupling reagent in the passive hemagglutination test. J. Immunol..

