# Supplementary material for: Leucine-Rich, Potent Anti-Bacterial Protein against Vibrio cholerae, Staphylococcus aureus from Solanum trilobatum Leaves
Source: Molecules. 2022 Feb 9;27(4):1167. doi: 10.3390/molecules27041167 (PMC8876335; doi:10.3390/molecules27041167)
Supplement: Supplementary file 1 [file molecules-27-01167-s001.zip › molecules-1569648-supplementary.pdf]

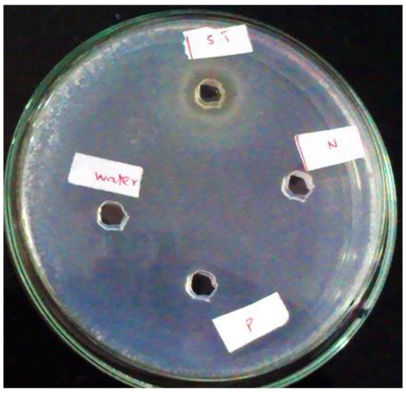

Figure S1. Proteolytic activity of solanum trilobatum aqueous extract where Casein was used as substrate by agar welldiffusion method: Top well: *Solanum trilobatum* (Crude extract), Bottom well Protein Extraction buffer (20mM Tris Buffer pH (6.4), Left well: Distilled water, Right well: 150 mM NaCl.

MATRIX SCIENCE **MASCOT Search Results**

Protein View: BIP8_TOBAC

Luminal-binding protein 8 (Fragment) OS=Nicotiana tabacum GN=BIP8 PE=2 SV=1

**Database:**	SwissProt
**Score:**	34
**Expect:**	13
**Nominal mass (M_r):**	32436
**Calculated pI:**	4.58
**Taxonomy:**	Nicotiana tabacum

Sequence similarity is available as an NCBI BLAST search of BIP8_TOBAC against nr.

Search parameters

**Enzyme:**	Trypsin: cuts C-term side of KR unless next residue is P.
**Fixed modifications:**	Carbamidomethyl (C)
**Variable modifications:**	Oxidation (M)
**Mass values searched:**	65
**Mass values matched:**	7

Protein sequence coverage: 33%

Matched peptides shown in ***bold red***.

```
  1 RIPKVQQLLK DYFDGKEPNK GVNPDEAVAY GAAVQGGILS GEGGDETKDI
 51 LLLDVAPLTL GIETVGGVMT KLIPRNTVIP SKKSQVFTTY QDQQTTVTIQ
101 VFEGERSLTK DCRLLGKFDL TGIAPAPRGT PQIEVTFEVD ANGILNVKAE
151 DKASGKSEKI TITNDKGRLS QEEIERMVKE AEEFAEEDKK VKERIDARNS
201 LETYVYNMRN QINDKDKLAD KLESDEKEKI ETATKEALEW LDDNQSAEKE
251 DYEEKLKEVE AVCNPIITAV YQKSGGAPGG ESGASEDDDH DEL
```

Unformatted sequence string: 293 residues (for pasting into other applications).

Sort peptides by ◉ Residue Number ○ Increasing Mass ○ Decreasing Mass

Show predicted peptides also

Start – End	Observed	Mr (expt)	Mr (calc)	ppm	M	Peptide
2 – 10	1066.6100	1065.6027	1065.6910	-82.8	1	R.IPKVQQLLK.D
2 – 16	1791.9340	1790.9267	1790.9931	-37.0	2	R.IPKVQQLLKDYFDGK.E
49 – 71	2384.2130	2383.2057	2383.3284	-51.5	0	K.DILLLDVAPLTLGIETVGGVMTK.L + Oxidation (M)
72 – 83	1365.7900	1364.7827	1364.8503	-49.5	2	K.LIPRNTVIPSKK.S
84 – 106	2705.4460	2704.4387	2704.2981	52.0	0	K.SQVFTTYQDQQTTVTIQVFEGER.S
118 – 128	1157.7100	1156.7027	1156.6241	68.0	0	K.FDLTGIAPAPR.G
177 – 189	1554.8060	1553.7987	1553.6919	68.8	1	R.MVKEAEEFAEEDK.K

Figure S2. Obtained peptide fragments of *Solanum trilobatum* antibacterial protein from Mass Spectrometer, identified the leucine richness and eight fragments, those fragments were matched with luminol binding protein of *Nicotiana tabacum*.
